# The Effect of COVID-19 Vaccination on Outpatient Antibiotic Prescribing in Older Adults: A Self-Controlled Risk-Interval Study

**DOI:** 10.1093/cid/ciae182

**Published:** 2024-05-03

**Authors:** Sarah C J Jorgensen, Kevin Brown, Anna E Clarke, Kevin L Schwartz, Colleen Maxwell, Nick Daneman, Jeffrey C Kwong, Derek R MacFadden

**Affiliations:** Clinical Epidemiology Program, Ottawa Hospital Research Institute, Ottawa, Ontario, Canada; Dalla Lana School of Public Health, University of Toronto, Toronto, Ontario, Canada; ICES (formerly Institute for Clinical Evaluative Sciences), Toronto, Ontario, Canada; Dalla Lana School of Public Health, University of Toronto, Toronto, Ontario, Canada; ICES (formerly Institute for Clinical Evaluative Sciences), Toronto, Ontario, Canada; Public Health Ontario, Toronto, Ontario, Canada; ICES (formerly Institute for Clinical Evaluative Sciences), Toronto, Ontario, Canada; Dalla Lana School of Public Health, University of Toronto, Toronto, Ontario, Canada; ICES (formerly Institute for Clinical Evaluative Sciences), Toronto, Ontario, Canada; Public Health Ontario, Toronto, Ontario, Canada; Unity Health Toronto, Li Ka Shing Knowledge Institute, Toronto, Ontario, Canada; ICES (formerly Institute for Clinical Evaluative Sciences), Toronto, Ontario, Canada; Schools of Pharmacy and Public Health Sciences, University of Waterloo, Waterloo, Ontario, Canada; ICES (formerly Institute for Clinical Evaluative Sciences), Toronto, Ontario, Canada; Division of Infectious Diseases, Sunnybrook Health Science Centre, Toronto, Ontario, Canada; Dalla Lana School of Public Health, University of Toronto, Toronto, Ontario, Canada; ICES (formerly Institute for Clinical Evaluative Sciences), Toronto, Ontario, Canada; Public Health Ontario, Toronto, Ontario, Canada; Centre for Vaccine Preventable Diseases, University of Toronto, Toronto, Ontario, Canada; Department of Family and Community Medicine, University of Toronto, Toronto, Ontario, Canada; University Health Network, Toronto, Ontario, Canada; Clinical Epidemiology Program, Ottawa Hospital Research Institute, Ottawa, Ontario, Canada; ICES (formerly Institute for Clinical Evaluative Sciences), Toronto, Ontario, Canada; Division of Infectious Diseases, University of Ottawa, Ottawa, Ontario, Canada

**Keywords:** COVID-19 vaccines, antibiotic prescribing, antibiotic stewardship, older adults

## Abstract

**Background:**

Coronavirus disease 2019 (COVID-19) vaccination has been associated with reduced outpatient antibiotic prescribing among older adults with laboratory-confirmed severe acute respiratory syndrome coronavirus 2 (SARS-CoV-2). We assessed the impact of COVID-19 vaccination on outpatient antibiotic prescribing in the broader population of older adults, regardless of SARS-CoV-2 infection status.

**Methods:**

We included adults aged ≥65 years who received their first, second, and/or third COVID-19 vaccine dose from December 2020 to December 2022. We used a self-controlled risk-interval design and included cases who received an antibiotic prescription 2–6 weeks before vaccination (pre-vaccination or control interval) or after vaccination (post-vaccination or risk interval). We used conditional logistic regression to estimate the odds of being prescribed (1) any antibiotic, (2) a typical “respiratory” infection antibiotic, or (3) a typical “urinary tract” infection antibiotic (negative control) in the post-vaccination interval versus the pre-vaccination interval. We accounted for temporal changes in antibiotic prescribing using background monthly antibiotic prescribing counts.

**Results:**

469 923 vaccine doses met inclusion criteria. The odds of receiving any antibiotic or a respiratory antibiotic prescription were lower in the post-vaccination versus pre-vaccination interval (aOR, .973; 95% CI, .968–.978; aOR, .961; 95% CI, .953–.968, respectively). There was no association between vaccination and urinary antibiotic prescriptions (aOR, .996; 95% CI, .987–1.006). Periods with high (>10%) versus low (<5%) SARS-CoV-2 test positivity demonstrated greater reductions in antibiotic prescribing (aOR, .875; 95% CI, .845–.905; aOR, .996; 95% CI, .989–1.003, respectively).

**Conclusions:**

COVID-19 vaccination was associated with reduced outpatient antibiotic prescribing in older adults, especially during periods of high SARS-CoV-2 circulation.

## Teaser

In this self-controlled risk-interval study, we found that COVID-19 vaccination was associated with reduced outpatient antibiotic prescribing among older adults. Moreover, our results suggest COVID-19 vaccination differentially reduces respiratory antibiotic prescriptions and benefits are accentuated during periods of high SARS-CoV-2 circulation.

Rising antibiotic resistance is an urgent threat to human health and has far-reaching negative social and economic repercussions [1–3]. Although antibiotic resistance impacts people of all ages, it is of particular concern for older adults, who have an elevated risk of antibiotic-resistant infections due to age-related declines in immune function, higher burdens of predisposing comorbidities, and more frequent exposure to resistant pathogens in healthcare settings [4]. Resources to combat antibiotic resistance have traditionally been directed toward antibiotic stewardship, infection prevention and control, and the development of new antibiotics and diagnostic tests [3, 5]. There is now growing recognition of the role of vaccines in reducing the emergence and spread of antibiotic resistance [6–8]. Vaccines can reduce antibiotic resistance through multiple pathways [8]. First, vaccines against bacterial infections directly limit antibiotic resistance by reducing the burden of infections caused by resistant bacteria [8–10]. Second, vaccines can eliminate the need for antibiotic therapy for infections caused by both resistant and susceptible bacteria, thereby reducing opportunities to select resistant variants [6–8]. Third, vaccines against viral infections can indirectly control antibiotic resistance by preventing secondary bacterial infections and by reducing presumptive symptom-based antibiotic prescribing [8, 10–12].

Patients with coronavirus disease 2019 (COVID-19) are frequently prescribed antibiotics [13, 14], despite evidence demonstrating that bacterial coinfection or secondary infection is rare [15]. Our team recently found that older adults with laboratory-confirmed severe acute respiratory syndrome coronavirus 2 (SARS-CoV-2) were most likely to receive an outpatient antibiotic prescription if they had risk factors for severe illness [14]. By contrast, prior completion of the primary COVID-19 vaccine series was associated with reduced antibiotic prescribing, suggesting that COVID-19 vaccination might reduce antibiotic prescribing by reducing the severity of infection [14]. The greatest population-wide impact of COVID-19 vaccination on antibiotic prescribing, however, would likely be by preventing infection rather than by reducing its severity.

In this study, we assessed the impact of COVID-19 vaccination on outpatient antibiotic prescribing in the broader population of older adults in Ontario, Canada, including those without laboratory-confirmed SARS-CoV-2, using a self-controlled risk-interval design study. A major advantage of this design is that it inherently controls for all measured and unmeasured time-invariant confounders, such as sex, race, socioeconomic status, and predisposing chronic medical conditions, because each case serves as their own control [16, 17]. Moreover, because vaccinated cases are compared with themselves over time, this design overcomes the challenge of identifying appropriate unvaccinated controls when vaccine coverage is high [18].

## METHODS

### Study Population and Setting

Ontario is Canada's most populous province with approximately 15.1 million residents, including 2.8 million residents aged 65 years and older, and a single-payer publicly funded healthcare system [19]. We included Ontario residents aged 65 years and older who received their first, second, and/or third COVID-19 vaccine dose between 14 December 2020 and 31 December 2022. We excluded those who died within 6 weeks after vaccination, had a hospital admission within 6 weeks before or after vaccination, were aged more than 110 years, or had an invalid birth date or death date.

### Data Sources

We used multiple health administrative databases that were linked using unique coded identifiers and analyzed at ICES (formerly Institute for Clinical Evaluative Sciences), an independent, nonprofit research institute whose legal status under Ontario's health information privacy law allows it to collect and analyze healthcare and demographic data, without consent, for health system evaluation and improvement. We extracted antibiotic prescription data from the Ontario Drug Benefit Program database, which contains information on all prescriptions dispensed to Ontario residents aged 65 years and older at outpatient pharmacies [20]. We obtained information on COVID-19 vaccinations from COVaxON, a central vaccine registry that contains complete information on all COVID-19 vaccinations administered in Ontario. Ontario's COVID-19 vaccination program began on 14 December 2020, with most residents receiving mRNA-based vaccines (Moderna Spikevax, Pfizer-BioNTech Comirnaty) [21, 22]. Due to supply constraints, the interval between the first and second doses of the primary vaccine series varied from 3 to 16 weeks. Third doses were available to people with immunosuppression in August 2021 and eligibility gradually expanded, based on risk, to include all adults aged 50 years and older on 13 December 2021. We obtained information on demographics and deaths from the Registered Persons Database. We determined the presence of comorbidities from multiple databases using validated algorithms that were based on diagnostic and procedure codes, as previously described [11, 23]. We identified residents of nursing homes using the Continuing Care Reporting System. We obtained information on hospital admissions using the Canadian Institute for Health Information Discharge Abstract Database. We identified temporal trends in SARS-CoV-2 test positivity and case counts using Public Health Ontario's Respiratory Virus Tool [24]. Details on all databases are provided in Supplementary Table 1.

### Statistical Analysis

**Figure 1.**
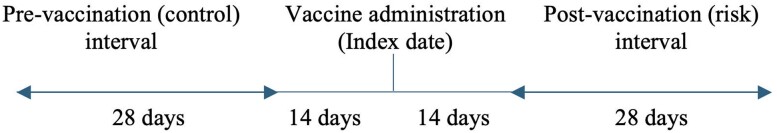
Self-controlled risk-interval study design.

We based the statistical analysis on the self-controlled risk-interval design (Figure 1) [16]. The null hypothesis with this design is that the risk of an event (ie, an antibiotic prescription) on an average day in the post-vaccination or risk interval is the same as the risk on an average day in the pre-vaccination or control interval [25]. The date of vaccination served as the index date. We defined the post-vaccination interval as 2 to 6 weeks after the index date and the pre-vaccination interval as 2 to 6 weeks before the index date. Because patients might delay vaccination after an acute illness/infection (ie, the healthy vaccinee effect) [26], we excluded the 2 weeks immediately before the index date from the pre-vaccination interval. We also excluded the 2 weeks immediately after the index date from the post-vaccination interval to allow time for immunity to develop in response to vaccination [27]. Only patients who received an antibiotic prescription during the pre-vaccination or post-vaccination interval were included in the analysis, with each patient serving as their own control. Those with a prescription in both the pre- and post-vaccination intervals did not contribute to the analysis. To obtain valid estimates using the self-controlled risk-interval design, events must be independent, conditional on time-varying covariates [28]. Although most antibiotic prescriptions during a short observation window would be for a single episode of infection, it is possible for the same patient to receive multiple sequential antibiotic prescriptions during a given window due to a recurrent or refractory infection (ie, non-independent prescriptions). To best accommodate this feature, we chose a dichotomous outcome, classified as either antibiotic exposure or no antibiotic exposure [12]. We used conditional logistic regression to estimate the odds ratio (OR) and corresponding 95% confidence interval (CI) of receiving an antibiotic prescription in the post-vaccination interval relative to the pre-vaccination interval [25, 29]. We performed analyses separately for each vaccine dose and pooled estimates across all doses. Because some people received more than 1 vaccine dose (up to a maximum of 3), we used a robust variance estimator to account for within-person correlation. We added a time-varying covariate corresponding to background monthly antibiotic prescription counts for Ontario residents aged 65 years and older to account for the potential differential risk of receiving an antibiotic prescription according to calendar time. We estimated the adjusted risk difference per 10 000 vaccine doses (aRD_10 000_) based on the adjusted OR (aOR) and baseline risk (see Schünemann et al [30] and Supplementary Methods). We repeated the analyses separately for respiratory antibiotics and urinary antibiotics; in each analysis, we adjusted for temporal changes in background respiratory and urinary antibiotic prescribing, respectively (Supplementary Table 2). The urinary antibiotics category served as a negative-control outcome (ie, no association expected). We examined effect modification by age (65–74, 75–84, ≥85 years), sex, nursing home residence, immunosuppression (organ or bone marrow transplant, human immunodeficiency virus [HIV] diagnosis, or other immunosuppressive condition or therapy), and periods of low (<5%) versus high (>10%) SARS-CoV-2 test positivity as a measure of population incidence. We performed all analyses using SAS, version 9.4 (SAS Institute).

## RESULTS

### Study Population

In total, 6 851 954 first, second, and third COVID-19 vaccine doses were administered to 2 522 122 Ontario residents aged 65 years and older between 14 December 2020 and 31 December 2022, of which 2 350 325, 2 349 430, and 2 152 199 were first, second, and third doses, respectively. Supplementary Figure 1 shows the flowchart for the selection of 469 923 (6.9%) vaccine doses, administered to 389 993 unique Ontario residents, who received an antibiotic prescription in either the pre- or post-vaccine interval and were included in the study. Baseline characteristics of the study population, by vaccine dose, are shown in Table 1. The mean (standard deviation) age was 75 (8) years, 229 211 (59%) were female, and 21 836 (5.6%) were nursing home residents. Patient characteristics were similar across vaccine doses. Compared with the source population, those included in the study were older, more likely to reside in a nursing home, and had a higher burden of comorbidity (Supplementary Table 3).

**Table 1.** Baseline Characteristics of Eligible Participants by COVID-19 Vaccine Dose

Variable	Any Vaccine Dose (n = 389 993)^a^	Vaccine Dose 1 (n = 156 177)	Vaccine Dose 2 (n = 157 092)	Vaccine Dose 3 (n = 156 654)
Age				
Mean ± SD, y	75.3 ± 7.9	75.6 ± 7.9	75.5 ± 7.9	74.9 ± 7.8
65–74 y	208 800 (53.5%)	82 033 (52.5%)	83 231 (53.0%)	86 577 (55.3%)
75–84 y	123 437 (31.7%)	49 903 (32.0%)	49 864 (31.7%)	48 541 (31.0%)
≥85 y	57 756 (14.8%)	24 241 (15.5%)	23 997 (15.3%)	21 536 (13.7%)
Sex				
Female	229 211 (58.8%)	92 820 (59.4%)	93 479 (59.5%)	92 683 (59.2%)
Male	160 782 (41.2%)	63 357 (40.6%)	63 613 (40.5%)	63 971 (40.8)
Comorbidities				
Diabetes mellitus	139 120 (35.7%)	56 872 (36.4%)	56 573 (36.0%)	55 064 (35.2%)
Asthma	76 546 (19.6%)	31 428 (20.1%)	30 980 (19.7%)	32 271 (20.6%)
COPD	49 719 (12.7%)	21 186 (13.6%)	20 600 (13.1%)	20 551 (13.1%)
Congestive heart failure	51 464 (13.2%)	21 578 (13.8%)	21 307 (13.6%)	20 095 (12.8%)
Liver disease	8346 (2.1%)	3402 (2.2%)	3429 (2.2%)	3347 (2.1%)
Chronic kidney disease	48 932 (12.5%)	20 723 (13.3%)	20 317 (12.9%)	19 208 (12.3%)
Immunosuppression	51 544 (13.2%)	21 445 (13.7%)	21 350 (13.6%)	21 658 (13.8%)
Dementia	41 366 (10.6%)	17 284 (11.1%)	17 382 (11.1%)	15 708 (10.0%)
Mental health diagnosis	120 880 (31.0%)	49 239(31.5%)	48 980 (31.2%)	49 103 (31.3%)
Charlson Comorbidity Index, median (IQR)	1 (0–2)^b^	1 (0–2)^c^	1 (0–2)^d^	1 (0–2)^e^
Nursing home resident	21 836 (5.6%)	9306 (6.0%)	9330 (5.9%)	8409 (5.4%)

Data are presented as n (%) unless otherwise stated.

Abbreviations: COPD, chronic obstructive pulmonary disease; COVID-19, coronavirus disease 2019; IQR, interquartile range; SD, standard deviation.

^a^Unique individuals. Individuals could have received >1 vaccine dose. For individuals who received >1 dose, age corresponds to age at first dose during the study period. Comorbidities and nursing home residence are reported as present if they were present at baseline for any dose.

^b^n = 117 249.

^c^n = 47 804.

^d^n = 47 753.

^e^n = 47 332.

### Antibiotic Prescriptions in the Post-Vaccination Interval Relative to the Pre-Vaccination Interval

**Figure 2.**
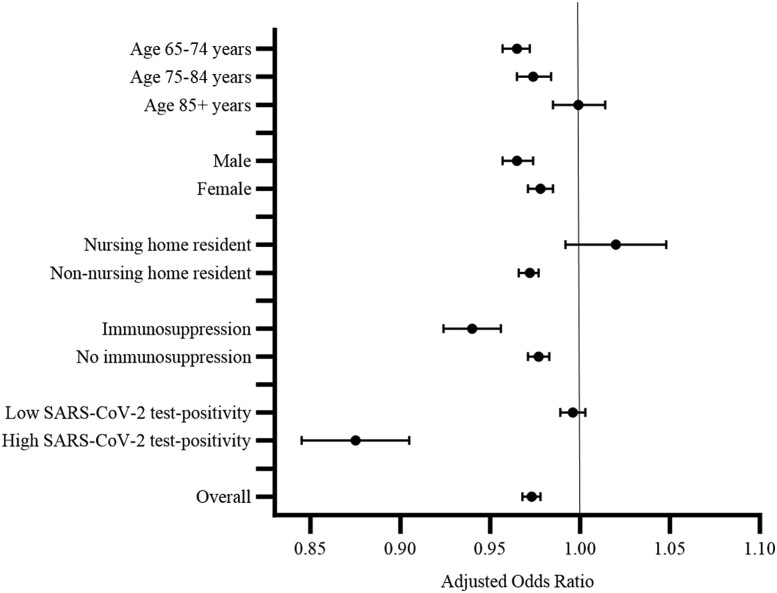
Adjusted odds ratios for antibiotic prescribing comparing the post-vaccination (risk) interval with the pre-vaccination (control) interval, by age, sex, nursing home residence, immunosuppression status, and Ontario SARS-CoV-2 test-positivity. Abbreviation: SARS-CoV-2, severe acute respiratory syndrome coronavirus 2.

Among vaccine recipients, 77 149 (3.3%), 79 971 (3.4%), and 73 156 (3.4%) were prescribed an antibiotic in the post-vaccination interval only following vaccine dose 1, 2, or 3, respectively. The proportion of people with a respiratory or urinary antibiotic prescription is shown in Table 2. The odds of receiving an antibiotic prescription were lower in the post-vaccination interval relative to the pre-vaccination interval in both the unadjusted analysis and the analysis adjusted for background monthly antibiotic prescription counts (OR = .961; 95% CI, .956–.966; aOR = .973; 95% CI, .968–.978, respectively) (Table 3). These estimates correspond to 9 (95% CI, 7–11) fewer antibiotic prescription episodes per 10 000 vaccine doses in the post-vaccination interval relative to the pre-vaccination interval. The odds of receiving a respiratory antibiotic prescription were also lower in the post-vaccination versus pre-vaccination interval (crude OR = .952; 95% CI, .945–.959; aOR = .961; 95% CI, .953–.968). The corresponding estimated reduction in respiratory antibiotic prescription episodes per 10 000 vaccine doses was 7 (95% CI, 6–9). There was no association between vaccination and urinary antibiotic prescriptions after accounting for background monthly urinary antibiotic prescription counts (crude OR; .987; 95% CI, .978–.997; aOR = .996; 95% CI, .987–1.006; aRD_10 000_ = 0; 95% CI −1, 2). As shown in Table 3, the aORs were lowest for vaccine dose 3 compared with dose 1 or 2. Results were consistent by sex and immunosuppression status (Figure 2, Supplementary Tables 4–6). The odds of receiving an antibiotic prescription were not lower in the post-vaccination versus pre-vaccination interval for those aged 85 years or older (aOR = .999; 95% CI, .985–1.014) or for nursing home residents (aOR = 1.020; 95% CI, .992–1.048), except for dose 1 for nursing home residents (aOR = .858; 95% CI, .795–.927) (Supplementary Table 4). The odds of receiving an antibiotic prescription in the post-vaccination interval compared with the pre-vaccination interval were lower during periods of high (>10%) versus low (<5%) SARS-CoV-2 test positivity (aOR = .875; 95% CI, .845–.905; aOR = .996; 95% CI, .989–1.003, respectively) (Figure 2, Supplementary Table 4). The corresponding reductions in antibiotic prescription episodes per 10 000 vaccine doses were 43 (95% CI, 32–53) and 1 (95% CI, −1, 4), respectively (Supplementary Table 4).

**Table 2.** Antibiotic Prescriptions in the Pre-Vaccination (Control) or Post-Vaccination (Risk) Interval for Eligible Participants by COVID-19 Vaccine Dose

	Vaccine Dose 1 (n = 2 350 325)	Vaccine Dose 2 (n = 2 349 430)	Vaccine Dose 3 (n = 2 152 199)
All antibiotics			
Pre- or post-vaccination interval	156 177 (6.6%)	157 092 (6.7%)	156 654 (7.3%)
Pre-vaccination interval	79 028 (3.4%)	77 121 (3.3%)	83 498 (3.9%)
Post-vaccination interval	77 149 (3.3%)	79 971 (3.4%)	73 156 (3.4%)
Respiratory antibiotics^a^			
Pre- or post-vaccination interval	84 122 (3.6%)	83 081 (3.5%)	90 609 (4.2%)
Pre-vaccination interval	42 320 (1.8%)	41 464 (1.8%)	48 270 (2.2%)
Post-vaccination interval	41 802 (1.8%)	41 617 (1.8%)	42 339 (2.0%)
Urinary antibiotics^b^			
Pre- or post-vaccination interval	53 337 (2.3%)	54 678 (2.3%)	52 416 (2.4%)
Pre-vaccination interval	26 846 (1.1%)	26 156 (1.1%)	27 722 (1.3%)
Post-vaccination interval	26 491 (1.1%)	28 522 (1.2%)	24 694 (1.2%)

Pre-vaccination (control) interval indicates 2 to 6 weeks before vaccination. Post-vaccination (risk) interval indicates 2 to 6 weeks after vaccination. Data are presented as n (%). The n values in the column headings represent the denominator and total number of unique individuals receiving a given vaccine dose, regardless of receipt of antibiotics.

Abbreviation: COVID-19, coronavirus disease 2019.

^a^Respiratory antibiotics: doxycycline, macrolides, penicillins, respiratory fluoroquinolones, and second- and third-generation cephalosporins.

^b^Urinary antibiotics: ciprofloxacin, fosfomycin, nitrofurantoin, trimethoprim, and trimethoprim/sulfamethoxazole.

**Table 3.** Unadjusted and Adjusted Effect Estimates of Antibiotic Prescribing Comparing the Post-Vaccination (Risk) Interval With the Pre-Vaccination (Control) Interval, by Antibiotic Grouping

Antibiotic Group	Post-Vaccination (Risk) Interval^a^	Pre-Vaccination (Control) Interval^a^	Unadjusted OR (95% CI)	Adjusted OR (95% CI)^b^	Adjusted Risk Difference Per 10 000 Vaccine Doses (95% CI)^b^
All antibiotics					
All doses	230 276/469 923 (49.0%)	239 647/469 923 (51.0%)	.961 (.956–.966)	.973 (.968–.978)	9 (7–11)
Dose 1	77 149/156 177 (49.4%)	79 028/156 177 (50.6%)	.976 (.967–.986)	.983 (.973–.993)	6 (2–9)
Dose 2	79 971/157 092 (50.9%)	77 121/157 092 (49.1%)	1.037 (1.027–1.047)	1.010 (.994–1.026)	−3 (−8–2)
Dose 3	73 156/156 654 (46.7%)	83 498/156 654 (53.3%)	.876 (.867–.885)	.909 (.897–.921)	34 (30–39)
Respiratory antibiotics^c^					
All doses	125 758/257 812 (48.8%)	132 054/257 812 (51.2%)	.952 (.945–.959)	.961 (.953–.968)	7 (6–9)
Dose 1	41 802/84 122 (49.7%)	42 320/84 122 (50.3%)	.988 (.974–1.001)	.993 (.980–1.007)	1 (−1–4)
Dose 2	41 617/83 081 (50.1%)	41 464/83 081 (49.9%)	1.004 (.990–1.017)	.997 (.983–1.013)	1 (−2–3)
Dose 3	42 339/90 609 (46.7%)	48 270/90 609 (53.3%)	.877 (.866–.889)	.898 (.885–.911)	22 (20–25)
Urinary antibiotics^d^					
All doses	79 707/160 431 (49.7%)	80 724/160 431 (50.3%)	.987 (.978–.997)	.996 (.987–1.006)	0 (−1–2)
Dose 1	26 491/53 337 (49.7%)	26 846/53 337 (50.3%)	.987 (.970–1.004)	.989 (.973–1.006)	1 (−1–3)
Dose 2	28 522/54 678 (52.2%)	26 156/54 678 (47.8%)	1.090 (1.072–1.109)	1.029 (.998–1.061)	−3 (−7–0)
Dose 3	24 694/52 416 (47.1%)	27 722/52 416 (52.9%)	.891 (.876–.906)	.958 (.934–.983)	5 (2–8)

Pre-vaccination (control) interval indicates 2 to 6 weeks before vaccination. Post-vaccination (risk) interval indicates 2 to 6 weeks after vaccination.

Abbreviations: CI, confidence interval; OR, odds ratio.

^a^Values are presented as n/N (%).

^b^Adjusted for background monthly antibiotic prescribing counts for Ontario residents ≥65 years.

^c^Respiratory antibiotics: doxycycline, macrolides, penicillins, respiratory fluoroquinolones, and second- and third-generation cephalosporins.

^d^Urinary antibiotics: ciprofloxacin, fosfomycin, nitrofurantoin, trimethoprim, and trimethoprim/sulfamethoxazole.

## DISCUSSION

We used a self-controlled risk-interval study design to demonstrate that COVID-19 vaccination is associated with reduced outpatient antibiotic prescribing among older adults. Moreover, our results suggest that COVID-19 vaccination differentially reduces prescriptions for antibiotics typically used for respiratory infections, and that the benefits of vaccination are accentuated during periods of high SARS-CoV-2 circulation. These findings suggest that COVID-19 vaccination could be an important tool to complement existing efforts to reduce antibiotic prescribing among older adults.

The plausibility of the study's findings is supported by several observations. First, effect sizes were greatest for antibiotics often prescribed for respiratory infections and were typically null for antibiotics often prescribed for urinary tract infections. Second, effect sizes were more pronounced during periods of high SARS-CoV-2 test positivity (in the general population). Likewise, effect sizes were greatest for vaccine dose 3 compared with doses 1 and 2 in the overall study population. This is likely explained by third dose availability in Ontario coinciding with high SARS-CoV-2 test positivity following the emergence and spread of the Omicron variant. Third, although there was no overall effect of vaccination on antibiotic prescribing among nursing home residents, the effect of the first dose specifically on antibiotic prescribing was large and significant. This likely reflects the high early COVID-19 case burden in nursing homes in Ontario, which coincided with the initial vaccine doses for nursing home residents. Taken together, our findings are compatible with vaccination reducing the burden of illness due to COVID-19 (via prevention of infection and/or reduction in illness severity) and corresponding presumptive antibiotic prescribing on an individual level.

The potential role of vaccination against COVID-19 and other respiratory viruses in reducing antibiotic resistance is of global interest. In 2021, the World Health Organization developed an Action Framework to guide vaccine stakeholders in leveraging vaccines to prevent and control antibiotic resistance [7]. Key objectives included increasing the collection and analysis of data to assess the impact of vaccines on antibiotic use and improving methodologies used for analysis of these data [7]. Although unnecessary antibiotic use in patients with COVID-19 is well documented [13], research on the impact of COVID-19 vaccines on antibiotic use is scarce. Our team recently reported the results of a population-based cohort study investigating antibiotic prescribing in older adults with laboratory-confirmed SARS-CoV-2 in the pre-Omicron era [14]. We found that prior completion of the primary COVID-19 vaccine series was associated with a 69% reduction in outpatient antibiotic prescribing rates among older adults with laboratory-confirmed SARS-CoV-2 compared with rates in unvaccinated older adults with laboratory-confirmed SARS-CoV-2 [14]. The present study expands on these findings in a number of important ways. First, it was not restricted to those with a documented SARS-CoV-2 infection, but instead assessed the broader potential impact of COVID-19 vaccines on outpatient antibiotic prescribing in older adults. Second, it extended into the Omicron-predominant era and included third vaccine doses. Last, unlike the cohort design, the self-controlled risk-interval design is not susceptible to bias due to noncomparability between vaccinated and unvaccinated populations [16, 17].

### Strengths and Limitations

This study has several strengths. First, we used deterministically linked, population-based databases within a universal healthcare system, which allowed us to identify all COVID-19 vaccinations and outpatient antibiotic prescriptions during the study period, thereby limiting potential selection bias. Second, detailed information on vaccination dates through a centralized COVID-19 vaccine registry minimized the potential for exposure misclassification bias. Third, the large study population allowed us to detect small differences in antibiotic prescribing in the post-vaccination (risk) interval relative to the pre-vaccination (control) interval, which could nevertheless translate into meaningful reductions in antibiotic use at the population level. Last, unlike cohort and case-control designs, the self-controlled risk-interval design inherently controls for time-fixed variables, which include difficult-to-measure potential confounders such as stable health-related behavior.

This study also has certain limitations. First, although the self-controlled risk-interval design is not susceptible to bias due to uncontrolled time-invariant confounding, it remains vulnerable to time-varying confounding [16, 17]. To mitigate the impact of time-varying confounding, we adjusted for background regional monthly antibiotic prescribing counts among older adults in Ontario. We also restricted the period of analysis to the 6 weeks before and after vaccination to limit temporal confounding due to fluctuations in local SARS-CoV-2 population incidence and infection-control interventions. However, the effectiveness of mRNA COVID-19 vaccines against infection and, to a lesser extent, severe illness wanes over time [31, 32]; a tradeoff of using a short risk interval was that we could not assess if the impact of the vaccines on antibiotic prescribing diminishes over time. Second, our analysis was restricted to adults aged 65 years and older who were prescribed an antibiotic either before or after vaccination. This population was older and had a larger proportion of nursing home residents and a higher burden of comorbidity than the general population of Ontario residents aged 65 years and older; results might not be generalizable to lower-risk older adults. Third, because many SARS-CoV-19 infections were not recorded following the upsurge in cases after the detection of the Omicron variant in Ontario, we were unable to assess effect modification by history of prior SARS-CoV-2 infection. Fourth, some people may have decreased their adherence to infection risk mitigation strategies following vaccination (ie, the “Peltzman effect”) [33, 34], in which case our results would be an underestimate of the total impact of vaccination on antibiotic prescribing, absent reverse risk compensation. Last, a shortcoming of the self-controlled risk-interval design in this context is that we could only obtain estimates of reductions in antibiotic prescriptions among vaccinees, which might under- or overestimate the impact of vaccination on antibiotic prescribing at the population level (ie, including both vaccine-exposed and -unexposed populations).

## Conclusions

In conclusion, the results of this study suggest that decreased outpatient antibiotic prescribing might be an important added benefit of COVID-19 vaccination, especially during periods of high SARS-CoV-2 test positivity. Although the impact of COVID-19 vaccination on antibiotic resistance remains uncertain, health systems wishing to decrease antibiotic use might consider COVID-19 vaccination as complementary to other infection-control strategies and stewardship efforts.

## Supplementary Data

Supplementary materials are available at *Clinical Infectious Diseases* online. Consisting of data provided by the authors to benefit the reader, the posted materials are not copyedited and are the sole responsibility of the authors, so questions or comments should be addressed to the corresponding author.

## Supplementary Material

ciae182_Supplementary_Data

## Notes

***Author Contributions.*** S. C. J. J. performed the data analysis and drafted the manuscript. A. E. C. performed the data extraction and data cleaning. D. R. M. obtained study funding. All authors contributed to the study concept and design and contributed to the critical review of the manuscript.

***Acknowledgments.*** Parts of these materials are based on data and information compiled and provided by the Ontario Ministry of Health (MOH) and the Canadian Institute for Health Information (CIHI). The authors thank Public Health Ontario for access to vaccination data from COVaxON and IQVIA Solutions Canada, Inc, for use of its Drug Information File. This document used data adapted from the Statistics Canada Postal Code^OM^ Conversion File, which is based on data licensed from Canada Post Corporation and/or data adapted from the Ontario Ministry of Health Postal Code Conversion File, which contains data copied under license from ©Canada Post Corporation and Statistics Canada.

***Disclaimer.*** The analyses, conclusions, opinions, results, and statements expressed herein are solely those of the authors and do not reflect those of the funding or data sources; no endorsement by ICES (formerly Institute for Clinical Evaluative Sciences), the MOH, the Ministry of Long-Term Care (MLTC), CIHI, or by the Ontario Health Data Platform (OHDP), its partners, or the Province of Ontario is intended or should be inferred.

***Data sharing.*** The dataset from this study is held securely in coded form at ICES. While legal data-sharing agreements between ICES and data providers (eg, healthcare organizations and government) prohibit ICES from making the dataset publicly available, access may be granted to those who meet prespecified criteria for confidential access, available at https://www.ices.on.ca/use-ices-data/ (email: das@ices.on.ca). The full dataset creation plan and underlying analytic code are available from the authors upon request, understanding that the computer programs may rely upon coding templates or macros that are unique to ICES and are therefore either inaccessible or may require modification.

***Financial support.*** This work was supported by ICES, which is funded by an annual grant from the Ontario Ministry of Health (MOH) and the Ministry of Long-Term Care (MLTC). This study also received funding from the Canadian Institutes of Health Research (CIHR). This study was supported by the Ontario Health Data Platform (OHDP), a Province of Ontario initiative to support Ontario's ongoing response to COVID-19 and its related impacts.
