# Supplementary material for: The Effect of COVID-19 Vaccination on Outpatient Antibiotic Prescribing in Older Adults: A Self-Controlled Risk-Interval Study
Source: Clin Infect Dis. 2024 May 3;79(2):375–81. doi: 10.1093/cid/ciae182 (PMC11327793; doi:10.1093/cid/ciae182)
Supplement: ciae182_Supplementary_Data [file ciae182_supplementary_data.pdf]

Supplemental Material

Supplement to: Jorgensen SCJ, *et al.* The effect of COVID-19 vaccination on outpatient antibiotic prescribing in older adults: a self-controlled risk-interval study.

Contents

Figure S1: Summary of case selection

Supplemental methods

Table S1: Linked health administrative databases

Table S2: Respiratory and urinary antibiotics

Table S3: Baseline characteristics of Ontario residents who received their first, second, and/or third COVID-19 vaccine dose, December 2020 to December 2022

Table S4: Antibiotic prescriptions in the post-vaccination (risk) interval relative to the pre-vaccination (control) interval around COVID-19 vaccination among older Ontario residents by age, sex, nursing home residence, immunosuppression status, and Ontario SARS-CoV-2 test-positivity, December 2020 to December 2022

Table S5: Respiratory antibiotic prescriptions in the post-vaccination (risk) interval relative to the pre-vaccination (control) interval around COVID-19 vaccination among older Ontario residents by age, sex, nursing home residence, immunosuppression status, and Ontario SARS-CoV-2 test-positivity, December 2020 to December 2022

Table S6: Urinary antibiotic prescriptions in the post-vaccination (risk) interval relative to the pre-vaccination (control) interval around COVID-19 vaccination among older Ontario residents by age, sex, nursing home residence, immunosuppression status, and Ontario SARS-CoV-2 test-positivity, December 2020 to December 2022

Figure S1: Summary of case selection

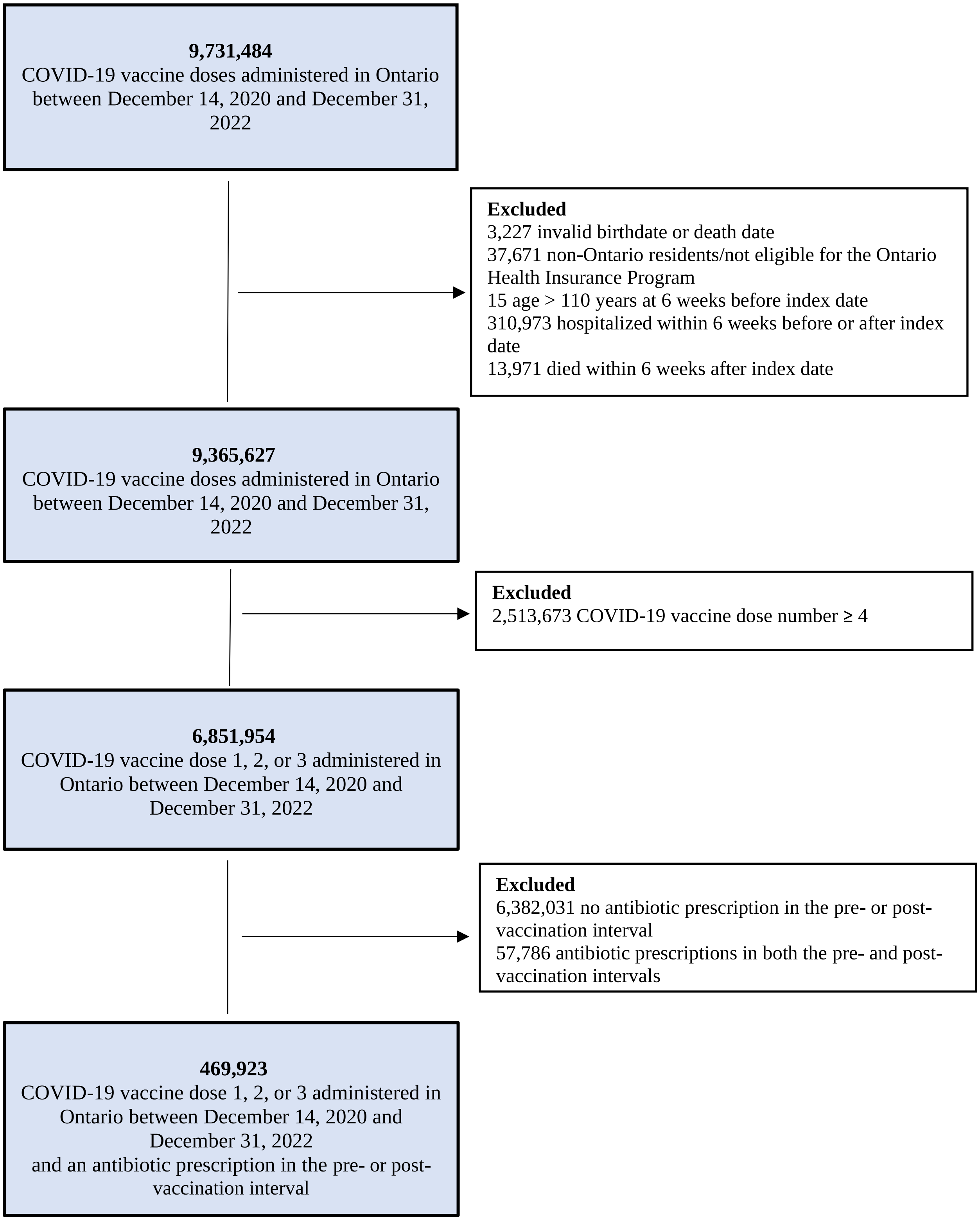

Supplemental methods

We computed the adjusted risk difference per 10,000 vaccine doses ($aRD_{10,000}$) from the adjusted odds ratio (aOR) using the following formula:[1]

$aRD_{10,000} = 10,000 * (CR – ((aOR*CR)/(1-CR+aOR*CR)))$

Where CR (control rate) represents the rate of antibiotic prescription episodes in the pre-vaccination (control) interval

We calculated confidence intervals by applying the above formula to the upper and lower confidence limits of the aOR.

1. Schünemann HJ, Vist GE, Higgins JPT, et al. Chapter 15: Interpreting results and drawing conclusions. In: Higgins JPT, Thomas J, Chandler J, et al (editors). *Cochrane Handbook for Systematic Reviews of Interventions* version 6.4 (updated August 2023). Cochrane, 2023. https://training.cochrane.org/handbook/current/chapter-15

Table S1: Linked health administrative databases

Database	Description
Registered Persons Database (RPDB)	Provincial database containing demographic information (e.g., date of birth, sex, postal code, and death date (if applicable)) for people eligible for health services in Ontario<br>Data is supplied by the Ministry of Health and enriched with ICES census derived data dating back to 1991
Canadian Institute for Health Information Discharge Abstract Database (CIHI-DAD)	National database containing demographic, clinical, and administrative information on all hospital discharges in Canada<br>Includes diagnostic and procedure codes based on ICD-10-CA codes and the Canadian Classification of Health Interventions dating back to 1988
Continuing Care Reporting System (CCRS)	National database containing demographic, clinical, functional, and resource use information on patients receiving care in chronic care hospitals or nursing homes in Canada dating back to 2003
Ontario Health Insurance Plan (OHIP)	Provincial database containing diagnostic and procedure information from inpatient and outpatient claims submitted by Ontario physicians, laboratories, and out-of-province physicians for Ontario residents dating back to 1991
Ontario Drug Benefit (ODB)	Provincial claims database for outpatient prescription drugs and vaccines (influenza) received under the ODB program for Ontario residents aged ≥65 years, residents of nursing homes, homes for special care, recipients of professional home services and social assistance, and the Trillium drug program
National Ambulatory Care Reporting System (NACRS)	National database containing information on all emergency department visits dating back to 2002
Ontario Mental Health Reporting System (OMHRS)	Provincial database with information on hospitalizations to designated psychiatric beds dating back to 2005
Canadian Institute for Health Information Same Day Surgery Database (CIHI-SDS)	National database containing information on day surgeries performed in Canada dating back to 1991
Postal Code Conversion File (PCCF)	A macro that links six-character postal codes to standard geographic areas such as dissemination area and census tracts
Ontario COVID-19 Vaccine Data (COVaxON)	A centralized COVID-19 vaccine information system compiled and managed by the Ministry of Health which contains comprehensive documentation (e.g., administration date, manufacturer, lot, DIN, location, and responsible public health unit) on all COVID-19 vaccination events in Ontario dating back to December 2020

Abbreviations: DIN = drug identification code; ICD-10-CA = Canadian version of the Internal Statistical Classification of Diseases and Health Related Problem, 10th Revision

Table S2: Respiratory and urinary antibiotics

Respiratory antibiotics	Urinary antibiotics
Doxycycline	Ciprofloxacin
Macrolides	Fosfomycin
Penicillins (± beta-lactamase inhibitor)	Nitrofurantoin
Respiratory fluoroquinolones	Trimethoprim
Second generation cephalosporins	Trimethoprim/sulfamethoxazole
Third generation cephalosporins	

Table S3: Baseline characteristics of Ontario residents who received their first, second, and/or third COVID-19 vaccine dose, December 2020 to December 2022

Characteristic	Source population N=2,522,122[a,b]	Study cohort N=389,993[a,b]
**Age (years)**		
Mean ± SD	74.2 ± 7.6	75.3 ± 7.9
65-74	1,494,808 (59.3%)	208,800 (53.5%)
75-84	728,288 (28.9%)	123,437 (31.7%)
≥85	299,026 (11.9%)	57,756 (14.8%)
**Sex**		
Female	1,369,383 (54.3%)	229,211 (58.8%)
Male	1,152,739 (45.7%)	160,782 (41.2%)
**Comorbidities**		
Diabetes mellitus	800,447 (31.7%)	139,120 (35.7%)
Asthma	359,206 (14.2%)	76,546 (19.6%)
COPD	202,956 (8.0%)	49,719 (12.7%)
Congestive heart failure	232,137 (9.2%)	51,464 (13.2%)
Liver disease	43,405 (1.7%)	8,346 (2.1%)
Chronic kidney diseases	233,559 (9.3%)	48,932 (12.5%)
Immunosuppression	236,420 (9.4%)	51,544 (13.2%)
Dementia	198,375 (7.9%)	41,366 (10.6%)
Mental health diagnosis	643,996 (25.5%)	120,880 (31.0%)
Charlson Comorbidity Index, median (IQR)	1 (0-2)[c]	1 (0-2)[d]
Nursing home resident	84,479 (3.3%)	21,836 (5.6%)

COPD: chronic obstructive pulmonary disease; IQR: interquartile range; SD: standard deviation

a. Number (%), unless otherwise stated
b. For individuals who received more than one dose, age corresponds to age at first dose during the study period. Comorbidities and nursing home residence are presented as present if they were present at baseline for any dose.
c. N=553,462
d. N=117,249

Table S4: Adjusted and unadjusted effect estimates of antibiotic prescribing comparing the post-vaccination (risk) interval relative to the pre-vaccination (control) interval, by age, sex, nursing home residence, immunosuppression status, and Ontario SARS-CoV-2 test-positivity

Antibiotic group	Post-vaccination (risk) interval[a,b]	Pre-vaccination (control) interval[b,c]	Unadjusted odds ratio (95% confidence interval)	Adjusted odds ratio (95% confidence interval)[d]	Adjusted risk difference per 10,000 vaccine doses (95% confidence interval)[d]
Age (years)					
65–74	**119,001/245,104 (48.6%)**	**126,103/245,104 (51.5%)**	**0.944 (0.937-0.951)**	**0.965 (0.957-0.972)**	**11 (9-13)**
Dose 1	40,014/82,033 (48.8%)	42,019/82,033 (51.2%)	0.952 (0.939-0.965)	0.967 (0.951-0.983)	10 (5-15)
Dose 2	41,322/81,826 (50.5%)	40,504/81,826 (49.5%)	1.020 (1.006-1.034)	0.984 (0.963-1.006)	5 (-2-11)
Dose 3	37,665/81,245 (46.4%)	43,580/81,245 (53.6%)	0.864 (0.852-0.876)	0.885(0.867-0.904)	40 (33-46)
75-84	**74,675/151,697 (49.2%)**	**77,022/151,697 (50.8%)**	**0.970 (0.960-0.979)**	**0.974 (0.965-0.984)**	**9 (6-12)**
Dose 1	24,887/49,903 (49.9%)	25,016/49,903 (50.1%)	0.995 (0.978-1.012)	0.991 (0.973-1.008)	3 (-3-9)
Dose 2	25,914/50,509 (51.3%)	24,595/50,509 (48.7%)	1.054 (1.035-1.072)	1.030 (1.000-1.061)	-10 (-20-0)
Dose 3	23,874/51,285 (46.6%)	27,411/51,285 (53.5%)	0.871 (0.856-0.886)	0.908 (0.887-0.929)	36 (38-45)
≥85	**36,600/73,122 (50.1%)**	**36,522/73,122 (50.0%)**	**1.002 (0.988-1.017)**	**0.999 (0.985-1.014)**	**0 (-6-6)**
Dose 1	12,248/24,241 (50.5%)	11,993/24,241 (49.5%)	1.021 (0.996-1.047)	1.022 (0.997-1.048)	-9 (-19-1)
Dose 2	12,735/24,757 (51.4%)	12,022/24,757 (48.6%)	1.059 (1.033-1.086)	1.060 (1.020-1.101)	-24 (-40- -8)
Dose 3	11,617/24,124 (48.2%)	12,507/24,124 (51.8%)	0.929 (0.906-0.953)	0.953 (0.927-0.980)	21 (9-32)
Sex					
Female	**137,168/278,982 (49.2%)**	**141,814/278,982 (50,8%)**	**0.967 (0.960-0.974)**	**0.978 (0.971-0.985)**	**8 (5-11)**
Dose 1	46,065/92,820 (49.6%)	46,755/92,820 (50.4%)	0.985 (0.973-0.998)	0.991 (0.978-1.004)	3 (-1-8)
Dose 2	47,710/93,479 (51.0%)	45,769/93,479 (49.0%)	1.042 (1.029-1.056)	1.019 (0.999-1.040)	-7 (-14-0)
Dose 3	43,393/92,683 (46.8%)	49,290/92,683 (53.2%)	0.880 (0.869-0.892)	0.913 (0.897-0.928)	35 (29-42)
Male	**93,108/190,941 (48.8%)**	**97,833/190,941 (51.2%)**	**0.952 (0.944-0.960)**	**0.965 (0.957-0.974)**	**11 (8-13)**
Dose 1	31,084/63,357 (49.1%)	32,273/63,357 (50.9%)	0.963 (0.948-0.978)	0.972 (0.957-0.988)	8 (4-13)
Dose 2	32,261/63,613 (50.7%)	31,352/63,613 (49.3%)	1.029 (1.013-1.045)	0.996 (0.972-1.021)	1 (-6-8)
Dose 3	29,763/63,971 (46.5%)	34,208/63,971 (53.5%)	0.870 (0.857-0.884)	0.903 (0.884-0.923)	33 (26-39)
Nursing home resident					
Yes	**11,342/22,455 (50.5%)**	**11,113/22,455 (49.5%)**	**1.021 (0.993-1.049)**	**1.020 (0.992-1.048)**	**-12 (-29-5)**
Dose 1	3,704/7,313 (50.7%)	3,609/7,313 (49.4%)	1.026 (0.980-1.074)	0.858 (0.795-0.927)	87 (45-126)
Dose 2	3,823/7,562 (50.6%)	3,739/7,562 (49.4%)	1.022 (0.977-1.070)	1.010 (0.948-1.077)	-6 (-47-32)
Dose 3	3,815/7,580 (50.3%)	3,765/7,580 (49.7%)	1.013 (0.969-1.060)	0.977 (0.928-1.028)	14 (-16-42)
No	**218,934/447,468 (48.9%)**	**228,534/447,468 (51.1%)**	**0.958 (0.953-0.963)**	**0.972 (0.966-0.977)**	**9 (8-11)**
Dose 1	73,445/148,864 (49.3%)	75,419/148,864 (50.7%)	0.974 (0.964-0.984)	0.982 (0.972-0.992)	6 (3-9)
Dose 2	76,148/149,530 (50.9%)	73,382/149,530 (49.1%)	1.038 (1.027-1.048)	1.008 (0.991-1.024)	-2 (-7-3)
Dose 3	69,341/149,074 (46.5%)	79,733/149,074 (53.5%)	0.870 (0.861-0.879)	0.897 (0.884-0.910)	38 (33-48)
Immunosuppression					
Yes	**23,813/49,329 (48.3%)**	**25,516/49,329 (51.7%)**	**0.933 (0.917-0.949)**	**0.940 (0.924-0.956)**	**30 (22-38)**
Dose 1	7,928/16,573 (47.8%)	8,645/16,573 (52.2%)	0.917 (0.890-0.945)	0.923 (0.894-0.952)	38 (24-52)
Dose 2	8,298/16,595 (50.0%)	8,297/16,595 (50.0%)	1.000 (0.970-1.031)	0.972 (0.933-1.013)	13 (-6-32)
Dose 3	7,587/16,161 (47.0%)	8,574/16,161 (53.1%)	0.885 (0.858-0.913)	0.925 (0.893-0.958)	41 (23-58)
No	**206,463/420,594 (49.1%)**	**214,131/420,594 (50.9%)**	**0.964 (0.959-0.970)**	**0.977 (0.971-0.983)**	**7 (6-9)**
Dose 1	69,221/139,604 (49.6%)	70,383/139,604 (50.4%)	0.983 (0.973-0.994)	0.991 (0.980-1.002)	3 (-1-6)
Dose 2	71,673/140,497 (51.0%)	68,824/140497 (49.0%)	1.041 (1.031-1.052)	1.016 (0.999-1.033)	-5 (-10-0)
Dose 3	65,569/140,493 (46.7%)	74,924/140,493 (53.3%)	0.875 (0.866-0.884)	0.905 (0.892-0.918)	34 (30-39)

Ontario SARS-CoV-2 test positivity					
Low (<5%)[e]	**155,133/309,323 (50.2%)**	**154,190/309,323 (49.9%)**	**1.006 (0.999-1.013)**	**0.996 (0.989-1.003)**	**1 (-1-4)**
High (>10%)[f]	**7,919/16,936 (46.8%)**	**9,017/16,936 (53.2%)**	**0.878 (0.852-0.905)**	**0.875 (0.845-0.905)**	**43 (32-53)**

a. Two to six weeks after vaccination
b. n/N (%)
c. Two to six weeks before vaccination
d. Adjusted for background monthly antibiotic prescribing counts for Ontario residents ≥ 65 years
e. January 17, 2021 – December 11, 2021
f. December 19, 2021 – May 14, 2022

Table S5: Adjusted and unadjusted effect estimates of respiratory antibiotic[a] prescribing comparing the post-vaccination (risk) interval relative to the pre-vaccination (control) interval, by age, sex, nursing home residence, immunosuppression status, and Ontario SARS-CoV-2 test-positivity

Antibiotic group	Post-vaccination (risk) interval[b,c]	Pre-vaccination (control) interval[c,d]	Unadjusted odds ratio (95% confidence interval)	Adjusted odds ratio (95% confidence interval)[e]	Adjusted risk difference per 10,000 vaccine doses (95% confidence interval)[e]
Age (years)					
65–74	**69,456/143,642 (48.4%)**	**74,186 /143,642 (51.7%)**	**0.936 (0.927-0.945)**	**0.952 (0.942-0.962)**	**9 (7-11)**
Dose 1	23,184/47,219 (49.1%)	24,035/47,219 (50.9%)	0.965 (0.947-0.982)	0.979 (0.958-1.000)	4 (0-7)
Dose 2	22,877/45,991 (49.7%)	23,114/45,991 (50.3%)	0.990 (0.972-1.008)	0.983 (0.964-1.003)	3 (-1-6)
Dose 3	23,395/50,432 (46.4%)	27,037/50,432 (53.6%)	0.865 (0.850-0.881)	0.888 (0.871-0.906)	24 (21-28)
75-84	**39,617/80,744 (49.1%)**	**41,127/80,744 (50.9%)**	**0.963 (0.950-0.976)**	**0.963 (0.950-0.976)**	**7 (5-10)**
Dose 1	13,122/25,991 (50.5%)	12,869/25,991 (49.5%)	1.020 (0.995-1.045)	1.009 (0.984-1.035)	-2 (-6-3)
Dose 2	13,133/26,062 (50.4%)	12,929/26,062 (49.6%)	1.016 (0.991-1.041)	1.007 (0.980-1.035)	-1 (-6-4)
Dose 3	13,362/28,691 (46.6%)	15,329/28,691 (53.4%)	0.872 (0.852-0.892)	0.887 (0.866-0.909)	25 (20-30)
≥85	**16,685/33,426 (49.9%)**	**16,741/33,426 (50.1%)**	**0.997 (0.976-1.018)**	**0.988 (0.967-1.010)**	**2 (-2-6)**
Dose 1	5,496/10,912 (50.4%)	5,416/10,912 (49.6%)	1.015 (0.977-1.054)	1.009 (0.971-1.048)	-2 (-9-5)
Dose 2	5,607/11,028 (50.8%)	5,421/11,028 (49.2%)	1.034 (0.996-1.074)	1.041 (0.997-1.088)	-8 (-16-1)
Dose 3	5,582/11,486 (48.6%)	5,904/11,486 (51.4%)	0.945 (0.911-0.981)	0.946 (0.912-0.981)	12 (4-19)
Sex					
Female	**68,085/139,502 (48.8%)**	**71,417/139,502 (51.2%)**	**0.953 (0.944-0.963)**	**0.960 (0.951-0.970)**	**7 (5-9)**
Dose 1	22,644/45,533 (49.7%)	22,889/45,533 (50.3%)	0.989 (0.971-1.008)	0.994 (0.975-1.012)	1 (-2-4)
Dose 2	22,423/44,921 (49.9%)	22,498/44,921 (50.1%)	0.997 (0.978-1.015)	0.990 (0.970-1.010)	2 (-2-5)
Dose 3	23,018/49,048 (46.9%)	26,030/49,048 (53.1%)	0.884 (0.869-0.900)	0.903 (0.886-0.921)	19 (15-22)
Male	**57,673/118,310 (48.8%)**	**60,637/118,310 (51.3%)**	**0.951 (0.941-0.962)**	**0.961 (0.951-0.972)**	**7 (5-9)**
Dose 1	19,158/38,589 (49.7%)	19,431/38,589 (50.4%)	0.986 (0.966-1.006)	0.993 (0.973-1.013)	1 (-2-5)
Dose 2	19,194/38,160 (50.3%)	18,966/38,160 (49.7%)	1.012 (0.992-1.033)	1.006 (0.984-1.029)	-1 (-5-3)
Dose 3	19,321/41,561 (46.5%)	22,240/41,561 (53.5%)	0.869 (0.852-0.886)	0.892 (0.873-0.911)	21 (17-25)
Nursing home resident					
Yes	**4,886/9,946 (49.1%)**	**5060/9,946 (50.9%)**	**0.966 (0.927-1.006)**	**0.964 (0.925-1.004)**	**10 (-1-21)**
Dose 1	1,568/3,341 (46.9%)	1,773/3,341 (53.1%)	0.884 (0.826-0.947)	0.676 (0.604-0.757)	101 (76-124)
Dose 2	1,659 (3,347 (49.6%)	1,688/3,347 (50.4%)	0.983 (0.918-1.052)	1.021 (0.935-1.115)	-6 (-33-19)
Dose 3	1,659/3258 (50.9%)	1,599/3,258 (49.1%)	1.038 (0.969-1.111)	0.929 (0.845-1.023)	18 (-6-40)
No	**120,872/247,866 (48.8%)**	**126,994/247,866 (51.2%)**	**0.952 (0.945-0.959)**	**0.961 (0.954-0.969)**	**7 (6-9)**
Dose 1	40,234/80,781 (49.8%)	40,547/80,781 (50.2%)	0.992 (0.979-1.006)	0.997 (0.983-1.011)	1 (-2-3)
Dose 2	39,958/79,734 (50.1%)	39,776/79,734 (49.9%)	1.005 (0.991-1.019)	0.996 (0.981-1.011)	1 (-2-3)
Dose 3	40,680/87,351 (46.6%)	46,671/87,351 (53.4%)	0.872 (0.860-0.883)	0.892 (0.879-0.905)	24 (21-26)
Immunosuppression					
Yes	**12,595/26,026 (48.4%)**	**13,431/26,026 (51.6%)**	**0.938 (0.916-0.960)**	**0.939 (0.917-0.961)**	**16 (11-22)**
Dose 1	4,131/8,522 (48.5%)	4,391/8,522 (51.5%)	0.941 (0.902-0.982)	0.942 (0.903-0.983)	15 (4-25)
Dose 2	4,236/8,536 (49.6%)	4,300/8,536 (50.4%)	0.985 (0.944-1.028)	0.988 (0.945-1.034)	3 (-9-14)
Dose 3	4,228/8,968 (47.2%)	4,740/8,968 (52.9%)	0.892 (0.856-0.930)	0.903 (0.866-0.941)	30 (18-41)
No	**113,163/231,786 (48.8%)**	**118,623/231,786 (51.2%)**	**0.954 (0.947-0.961)**	**0.963 (0.956-0.971)**	**7 (5-8)**
Dose 1	37,671/75,600 (49.8%)	37,929/75,600 (50.2%)	0.993 (0.979-1.007)	0.999 (0.985-1.014)	0 (-2-3)
Dose 2	37,381/74,545 (50.2%)	37,164/74,545 (49.9%)	1.006 (0.992-1.020)	0.998 (0.982-1.014)	0 (-2-3)
Dose 3	38,111/81,641 (46.7%)	43,530/81,641 (53.3%)	0.876 (0.864-0.888)	0.896 (0.882-0.909)	22 (19-25)

Ontario SARS-CoV-2 test positivity					
Low (<5%)[f]	**82,700/165,376 (50.0%)**	**82,676/165,323 (50.0%)**	**1.00 (0.991-1.009)**	**0.996 (0.987-1.005)**	**1 (-1-2)**
High (>10%)[g]	**4,574/10,483 (43.6%)**	**5,909/10,483 (56.4%)**	**0.774 (0.745-0.805)**	**0.804 (0.765-0.844)**	**45 (35-53)**

a. Respiratory antibiotics: Doxycycline, macrolides, penicillins, respiratory fluoroquinolones, and second and third generation cephalosporins
b. Two to six weeks after vaccination
c. n/N (%)
d. Two to six weeks before vaccination
e. Adjusted for background monthly respiratory antibiotic prescribing counts for Ontario residents ≥ 65 years
f. January 17, 2021 – December 11, 2021
g. December 19, 2021 – May 14, 2022

Table S6: Adjusted and unadjusted effect estimates of urinary antibiotic[a] prescribing comparing the post-vaccination (risk) interval relative to the pre-vaccination (control) interval, by age, sex, nursing home residence, immunosuppression status, and Ontario SARS-CoV-2 test-positivity, December 2020 to December 2022

Antibiotic group	Risk/post-vaccination interval[b,c]	Control/pre-vaccination interval[c,d]	Unadjusted odds ratio (95% confidence interval)	Adjusted odds ratio (95% confidence interval)[e]	Adjusted risk difference per 10,000 vaccine doses (95% confidence interval)[e]
Age (years)					
65–74	**36,587/74,229 (49.3%)**	**37,642/74,229 (50.7%)**	**0.972 (0.959-0.985)**	**0.997 (0.983-1.011)**	**0 (-1-2)**
Dose 1	12,226/25,039 (48.8%)	12,813/25,039 (51.2%)	0.954 (0.931-0.978)	0.969 (0.943-0.996)	3 (0-5)
Dose 2	13,221/25,354 (52.2%)	12,133/25,354 (47.9%)	1.090 (0.063-1.117)	1.003 (0.960-1.048)	0 (-5-4)
Dose 3	11,140/23,836 (46.7%)	12,696/23,836 (53.3%)	0.877 (0.855-0.900)	0.948 (0.908-0.990)	5 (1-8)
75-84	**27,620/54,781 (49.8%)**	**27,521/54,781 (50.2%)**	**0.991 (0.974-1.007)**	**0.991 (0.975-1.008)**	**1 (-1-3)**
Dose 1	8,936/17,897 (49.9%)	8,961/17,897 (50.1%)	0.997 (0.968-1.027)	0.987 (0.957-1.017)	2 (-2-5)
Dose 2	9,709/18,583 (52.3%)	8,874/18,583 (47.8%)	1.094 (1.063-1.126)	1.043 (0.986-1.103)	-6 (-14-2)
Dose 3	8,615/18,301 (47.1%)	9,686/18,301 (52.9%)	0.889 (0.864-0.916)	0.979 (0.936-1.025)	3 (-3-8)
≥85	**15,860/31,421 (50.5%)**	**15,561/31,421 (49.5%)**	**1.019 (0.997-1.042)**	**1.016 (0.993-1.039)**	**-3 (-7-1)**
Dose 1	5,329/10,401 (51.2%)	5,072/10,401 (48.8%)	1.051 (1.011-1.092)	1.054 (1.014-1.096)	-10 (-18- -3)
Dose 2	5,592/10,741 (52.1%)	5,149/10,741 (47.9%)	1.086 (1.046-1.128)	1.063 (0.993-1.137)	-12 (-26-1)
Dose 3	4,939/10,279 (48.1%)	5,340/10,279 (52.0%)	0.925 (0.890-0.961)	0.952 (0.909-0.997)	9 (1-16)
Sex					
Female	**56,778/113,613 (50.0%)**	**56,835/113,613 (50.0%)**	**0.999 (0.988-1.010)**	**1.007 (0.996-1.019)**	**-1 (-3-1)**
Dose 1	18,915/37810 (50.0%)	18,895 (50.0%)	1.001 (0.981-1.021)	1.003 (0.930-1.024)	0 (-3-10)
Dose 2	20,387/38,842 (52.5%)	18,455/38,842 (47.5%)	1.105 (1.083-1.127)	1.064 (1.025-1.105)	-9 (-15- -4)
Dose 3	17,476/36,961 (47.3%)	19,485/36,961 (52.7%)	0.897 (0.879-0.915)	0.961 (0.932-0.992)	6 (1-11)
Male	**22,929/46,818 (49.0%)**	**23,889/46,818 (51.0%)**	**0.960 (0.943-0.977)**	**0.970 (0.953-0.987)**	**2 (1-4)**
Dose 1	7,576/15,527 (48.8%)	7,951/15,527 (51.2%)	0.953 (0.923-0.983)	0.956 (0.926-0.987)	3 (1-5)
Dose 2	8,135/15,836 (51.4%)	7,701/15,836 (48.6%)	1.056 (1.024-1.090)	0.957 (0.906-1.010)	3 (-1-7)
Dose 3	7,218/15,455 (46.7%)	8,237/15,455 (53.3%)	0.876 (0.849-0.904)	0.951 (0.907-0.997)	4 (0-8)
Nursing home resident					
Yes	**5,508/10,752 (51.2%)**	**5,244/10,752 (48.8%)**	**1.050 (1.010-1.092)**	**1.049 (1.009-1.091)**	**-14 (-27- -3)**
Dose 1	1,782/3,366 (52.9%)	1,584/3,366 (47.1%)	1.125 (1.051-1.204)	1.035 (0.931-1.151)	-10 (-42-19)
Dose 2	1,850/3,616 (51.2%)	1,766/3,616 (48.8%)	1.048 (0.981-1.118)	0.962 (0.868-1.066)	11 (-20-39)
Dose 3	1,876/3,770 (49.8%)	1,894/3,770 (50.2%)	0.990 (0.929-1.056)	0.985 (0.923-1.050)	5 (-15-24)
No	**74,199/149,679 (49.6%)**	**75,480/149,679 (50.4%)**	**0.983 (0.973-0.993)**	**0.993 (0.983-1.003)**	**1 (0-2)**
Dose 1	24,709/49,971 (49.5%)	25,262/49,971 (50.6%)	0.978 (0.961-0.995)	0.979 (0.962-0.996)	2 (0-4)
Dose 2	26,672/51,062 (52.2%)	24,390/51,062 (47.8%)	1.094 (1.075-1.113)	1.034 (1.001-1.068)	-4 (-7-0)
Dose 3	22,818/48,646 (46.9%)	25,828/48,646 (53.1%)	0.883 (0.868-0.899)	0.953 (0.926-0.981)	6 (2-9)
Immunosuppression					
Yes	**9,208/19,072 (48.3%)**	**9,864/19,072 (51.7%)**	**0.934 (0.908-0.960)**	**0.936 (0.911-0.963)**	**13 (7-18)**
Dose 1	3,013/6,424 (46.9%)	3,411/6,424 (53.1%)	0.883 (0.841-0.928)	0.885 (0.843-0.930)	23 (14-31)
Dose 2	3,292/6491 (50.7%)	3,199/6,491 (49.3%)	1.029 (0.980-1.080)	0.942 (0.878-1.011)	11 (-2-23)
Dose 3	2,903/6,157 (47.2%)	3,254/6,157 (52.3%)	0.892 (0.849-0.938)	0.935 (0.879-0.995)	14 (1-26)
No	**70,499/141,359 (49.9%)**	**70,860/141,359 (50.1%)**	**0.995 (0.985-1.005)**	**1.005 (0.994-1.015)**	**-1 (-2-1)**
Dose 1	23,478/46,913 (50.1%)	23,435/46,913 (50.0%)	1.002 (0.984-1.020)	1.004 (0.986-1.023)	0 (-2-1)
Dose 2	25,230/48,187 (52.4%)	22,957/48,187 (47.6%)	1.099 (1.080-1.119)	1.050 (1.015-1.087)	-5 (-9- -2)
Dose 3	21,791/46,259 (47.1%)	24,468/46,259 (52.9%)	0.891 (0.874-0.907)	0.963 (0.936-0.991)	4 (1-8)

Ontario SARS-CoV-2 test positivity					
Low (<5%)[f]	**54,221/106,709 (50.8%)**	**52,488/106,709 (49.2%)**	**1.033 (1.021-1.1.045)**	**1.002 (0.990-1.015)**	**0 (-2-1)**
High (>10%)[g]	**2,638/5,187 (50.9%)**	**2,549/5,187 (49.1%)**	**1.035 (0.980-1.093)**	**1.038 (0.981-1.098)**	**-4 (-10-2)**

a. Urinary antibiotics: Ciprofloxacin, fosfomycin, nitrofurantoin, trimethoprim, and trimethoprim/sulfamethoxazole
b. Two to six weeks after vaccination
c. n/N (%)
d. Two to six weeks before vaccination
e. Adjusted for background monthly urinary antibiotic prescribing counts for Ontario residents ≥ 65 years
f. January 17, 2021 – December 11, 2021
g. December 19, 2021 – May 14, 2022
